# Intrafascial versus interfascial nerve sparing in radical prostatectomy for localized prostate cancer: a systematic review and meta-analysis

**DOI:** 10.1038/s41598-017-11878-7

**Published:** 2017-09-13

**Authors:** Hong Weng, Xian-Tao Zeng, Sheng Li, Xiang-Yu Meng, Ming-Jun Shi, Da-Lin He, Xing-Huan Wang

**Affiliations:** 1 Department of Urology, Zhongnan Hospital of Wuhan University, Wuhan, 430071 China; 2 Center for Evidence-Based and Translational Medicine, Zhongnan Hospital of Wuhan University, Wuhan, 430071 China; 3 Institut Curie, Centre National de la Recherche Scientifique (CNRS), Unité Mixte de Recherche 144, Paris, 75248 France; 4 Department of Urology, The First Affiliated Hospital of Xi’an Jiaotong University, Xi’an, 710061 China

## Abstract

The present study aimed to systematically evaluate the effectiveness and safety of the intrafascial and interfascial nerve sparing (ITR-NS and ITE-NS) radical prostatectomy. PubMed, Embase, and Cochrane Library databases were searched for eligible studies. Meta-analysis with random-effects model was performed. Six comparative trials were selected and embraced in this research, including one randomized controlled trial, three prospective comparative trials, and two retrospective comparative trials. With regard to perioperative parameters, no significant association of operative time, blood loss, transfusion rates, duration of catheterization, and hospital stay existed between ITR-NS and ITE-NS. With respect to the functional results, ITR-NS had advantages in terms of both continence and potency recovery compared with ITE-NS. In reference to the oncologic results, the ITR-NS showed lower overall positive surgical margin (PSM) compared with ITE-NS but pT2 PSM and biochemical recurrence free rates were similar to the two surgical types. This study demonstrates that ITR-NS has better continence at 6 mo and 36 mo and better potency recovery at 6 mo and 12 mo postoperatively, regardless of the surgical technique. The cancer control of ITR-NS was also better than that of ITE-NS. This may be explained by the fact that patients in ITE-NS group present higher risk cancer than patients in ITR-NS group.

## Introduction

Prostate cancer is the most common nonskin malignancy in western men and the second leading cause of cancer-related death among men in United States^1, 2^. Radical prostatectomy (RP) is the standard surgical treatment for localized prostate cancer. However, the postoperative impotence and especially the urinary incontinence following RP are still a matter of trouble for patients^3–5^.

Many approaches, such as open retropubic RP, laparoscopy RP, and robot-assisted RP, have been applied in RP. Recently, Reeves *et al*.^6^ performed a systematic review and meta-analysis that summarized the existing evidence on the influence of the preservation of the NVBs on continence after RP. Their study suggested that early urinary continence rate (at 6 mo time point) was improved for patients undergoing nerve-sparing RP compared with patients undergoing non-nerve-sparing RP. In recent years, certain urologists have compared intrafascial nerve-sparing (ITR-NS) with interfascial nerve-sparing (ITE-NS) RP, and the results are inconclusive^7–12^. The ITR-NS technique is considered a dissection that follows a plane on the prostate capsule and it allows a whole-thickness preservation of the NVBs^13^. Reeves and colleagues did not assess the specific nerve-sparing technique^6^.

To answer this important question, we carried out the present systematic review and meta-analysis to summarize the current existing evidence for clinical practice. In this study, we comprehensively evaluated the functional outcomes, oncologic outcomes, and perioperative parameters of ITR-NS and ITE-NS.

## Methods

This systematic review and meta-analysis was performed according to the Preferred Reporting Items of Systematic Reviews and Meta-analysis (PRISMA) statement^14^. The study protocol of this systematic review and meta-analysis was published in the PROSPERO register (registration number: CRD42016038687).

### Search strategy

A literature search of the PubMed, Embase, and Cochrane Library was conducted up to March 2016 (updated to July 2017) to identify potentially relevant trials. The following terms were searched: (“prostatic neoplasms” OR “prostate” OR “prostate cancer”) AND (“prostatectomy” OR “radical prostatectomy”) AND (“interfascial” OR “intrafascial”). The study language was restricted to English. In addition, reference lists in the recent reviews, meta-analysis, and included articles were checked for identifying any potentially relevant studies. The detailed search strategy for each database is presented in Supplementary Table 1.

### Eligibility criteria

The inclusion criteria were established according to PICOS (patients, intervention, comparison, outcomes, and study design) principle as presented in Table 1. The exclusion criteria were as following: duplicated studies, single cohort studies (i.e. studies without comparison groups), case-control studies and cross-sectional studies were excluded.

**Table 1:** Inclusion criteria of the systematic review and meta-analysis.

Criteria	Description
Patients	Adult men who underwent radical prostatectomy for prostate cancer
Intervention	ITR-NS, which was defined as the preservation of the periprostatic fascia and nerves by cutting adjacent prostate and dissecting the plane between prostatic capsule and prostatic fascia
Comparison	ITE-NS was the control group, which was defined as the dissection of the plane between prostatic fascia and endopelvic fascia; studies were not be selected or excluded based on surgical approaches (i.e. retropubic, laparoscopic, and robotic approaches)
Primary outcomes	Functional and oncologic results. The functional results included postoperative urinary continence rate and potency recovery rate, and the oncologic results included PSM, pT2 PSM, and BCR free rates
Secondary outcomes	Perioperative parameters (i.e. operative time, blood loss, transfusion rates, duration of catheterization, and hospital stay)
Study design	RCTs or longitudinal controlled studies were included (i.e. RCTs, prospective or retrospective cohort comparative studies)

PSM = positive surgical margin; BCR = biochemical recurrence; RCTs = randomized controlled trials.

### Study selection and data extraction

Two authors independently screened titles and abstracts of all search results. Studies were selected based on the pre-specified inclusion and exclusion criteria. Any discrepancy was resolved by discussion. Two authors independently extracted the following data from each identified study: study details (name of first author, country, year, contact details, conflicts of interest), methods (study design, duration, clinical setting), patients (sample size, baseline characteristics), intervention (surgical approach, comparison group), and outcome (postoperative urinary continence rate, potency recovery rate, PSM, pT2 PSM, BCR free rates, operative time, blood loss, transfusion rates, duration of catheterization, and hospital stay).

### Risk of bias and quality of evidence assessment

We used the Cochrane collaboration’s tool for risk of bias assessment of RCTs and the Newcastle-Ottawa Scale for risk of bias assessment of observational studies^15–17^. The Cochrane collaboration’s tool assesses risk of bias in six domains: (1) selection bias; (2) performance bias; (3) detection bias; (4) attribution bias; (5) reporting bias; and (6) other bias^15^. The Newcastle-Ottawa Scale assesses risk of bias in three domains: (1) selection of the study population; (2) comparability of groups; and (3) ascertainment of outcome^16^. We evaluated that follow-up was adequate if the maximum follow-up was more than 2 yr (i.e. 24 mo). The quality of evidence was assessed according to the GRADE system using GRADEpro GDT software.

### Statistical analysis

Meta-analysis was performed to aggregate the results if studies were sufficiently similar. Due to the clinical heterogeneity implicated in the included studies, random-effects model was applied to estimate summary risk ratios (RRs) and corresponding 95% confidence intervals (CIs). Sensitivity analysis was conducted through sequentially excluding retrospective studies. Subgroup analysis according to timing of outcome measurement was performed if sufficient data was available. Heterogeneity was tested using chi-square (*p* ≤ 0.1) test and I^2^ metric. All statistical analysis was performed using RevMan 5.3 (Cochrane Collaboration, Oxford, UK). A two-sided *p* value less than 0.05 represented a statistically significant difference, except for heterogeneity test. Publication bias was detected using funnel plot if the included studies were more than five for each outcome.

## Results

### Literature search and study characteristics

**Figure 1:**
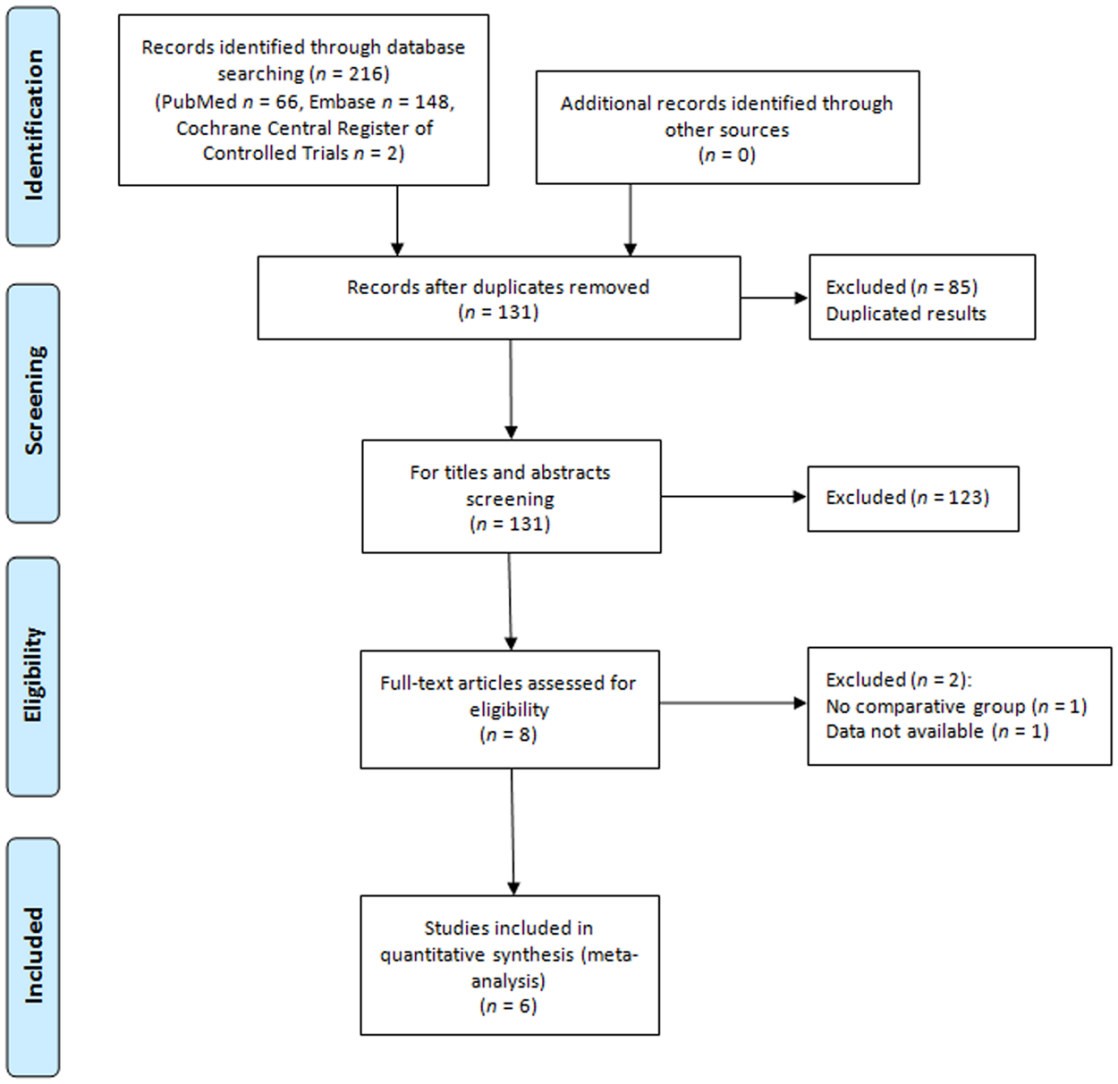
Flow diagram of the systematic review and meta-analysis.

Figure 1 shows the PRISMA flowchart of the systematic review and meta-analysis. Our search initially yielded a total of 216 records. After exclusion of duplicate articles, 131 records were screened through titles and abstracts. Finally, 6 studies involving 1663 patients (ITR-NS: 916 patients, ITE-NS: 747 patients) were included in this systematic review and meta-analysis^7–12^.

The characteristics of included studies are presented in Table 2. These six comparative trials included one RCT^7^, three prospective comparative trials^8, 9, 12^, and two retrospective comparative trials^10, 11^. There were two studies^7, 9^ using laparoscopic RP, two studies^8, 11^ involving robot-assisted RP, and two studies^10, 12^ applying open retropubic RP. The definition of the ITR-NS and ITE-NS in the included studies is presented in Supplementary Table 2. Study sample sizes ranged from 41^8^ and 420^12^. Studies were published from 2010 to 2015 in Europe^7, 10–12^ and Asia^8, 9^. All the studies used bilateral nerve-sparing radical prostatectomy technique.

**Table 2:** Characteristics of included studies.

Reference	Study	Surgical approach	No. of cases, type	Age, yr	PSA, ng/ml	Size of prostate, ml	pT2, %	Gleason score 4–6, %	Gleason score 7, %	Gleason score 8–10, %
Stolzenburg^7^	RCT	Laparoscopic	200 ITR-NS	60 (41–73)	6 (1.0–31)	40 (20–105)	89	51.5	24.5	21
			200 ITE-NS	62 (41–75)	6.8 (0.6–24)	44.5 (16–166)	81	45.9	30.1	24
Ko^8^	Prospective	RARP	9 ITR-NS	52.44 ± 5.38	4.96 ± 1.26	49.94 ± 12.66	100	22.2	77.8	0
			32 ITE-NS	59.05 ± 6.95	5.28 ± 2.17	59.09 ± 18.61	100	28.1	71.9	0
Zheng^9^	Prospective	Laparoscopic	65 ITR-NS	65 (56–70)	5.12 (2.90–7.85)	—	86	—	—	0
			130 ITE-NS	65 (55–69)	5.98 (2.98–8.06)	—	80	—	—	0
Khoder^10^	Retrospective	Retropubic	203 ITR-NS	62.7 (35.9–82.1)	5.6 (0.3–9.9)	—	93	66.4	32.7	0.9
			163 ITE-NS	63.5 (41.1–77.6)	7.0 (0.6–15.0)	—	82	40.8	54.6	4.6
Ihsan-Tasci^11^	Retrospective	RARP	200 ITR-NS	60.8 ± 6.5^a^	8.6 ± 3.2^a^	41.5 ± 12.4^a^	91	35	22.8	2.1
			41 ITE-NS				15	7.5	4.8	0
Khoder^12^	Prospective	Retropubic	239 ITR-NS	68.0 (48.2–81.9)	5.9 (0.3–9.9)	46 (7–160)	—	—	—	—
			181 ITE-NS	68.1 (48.1–80.7)	8.2 (0.1–95.0)	44 (14–148)	—	—	—	—

RCT = randomized controlled trial; RARP = robot-assisted radical prostatectomy; PSA = prostate specific antigen; ITR-NS = intrafacial nerve sparing; ITE-NS = interfacial nerve sparing.

^a^Mean ± sd of the total patients in the two groups.

### Risk of bias assessment

Tables 3 and 4 showed the risk of bias assessment of included studies. The random sequence generation, allocation concealment, and blinding of the RCT were all unclear (Table 3). Therefore, the risk of bias of the RCT was unclear. The majority of the longitudinal controlled studies were considered to have low to moderate risk of bias (Table 4). Three studies^7, 10, 11^ had the proportion of high Gleason score (8–10 score). Patients in one study^8^ who underwent RP were relatively younger than those in other studies included in this meta-analysis. Furthermore, the surgical techniques used in the included studies were different. These factors would introduce some selection bias and clinical heterogeneity.

**Table 3:** Risk of bias assessment of randomized controlled trial included in the meta-analysis.

Study	Random sequence generation	Allocation concealment	Blinding of participants and personnel	Blinding of outcome assessment	Incomplete outcome data	Selective reporting	Other bias
Stolzenburg^7^	Unclear	Unclear	Unclear	Unclear	Low	Low	Unclear

**Table 4:** Risk of bias assessment of observational studies included in the meta-analysis.

Study	Selection			Comparability	Outcome		
	Representativeness of exposed cohort	Selection of nonexposed	Ascertainment of exposure		Assessment of outcome	Adequate follow-up length	Adequacy of follow-up
Ko^8^	★	★	★	★★	★	☆	★
Zheng^9^	★	★	★	★★	★	☆	★
Khoder^10^	★	★	★	★★	★	☆	★
Ihsan-Tasci^11^	★	★	★	★★	★	☆	★
Khoder^12^	★	★	★	★★	★	★	★

### Primary outcomes

#### Urinary continence

**Figure 2:**
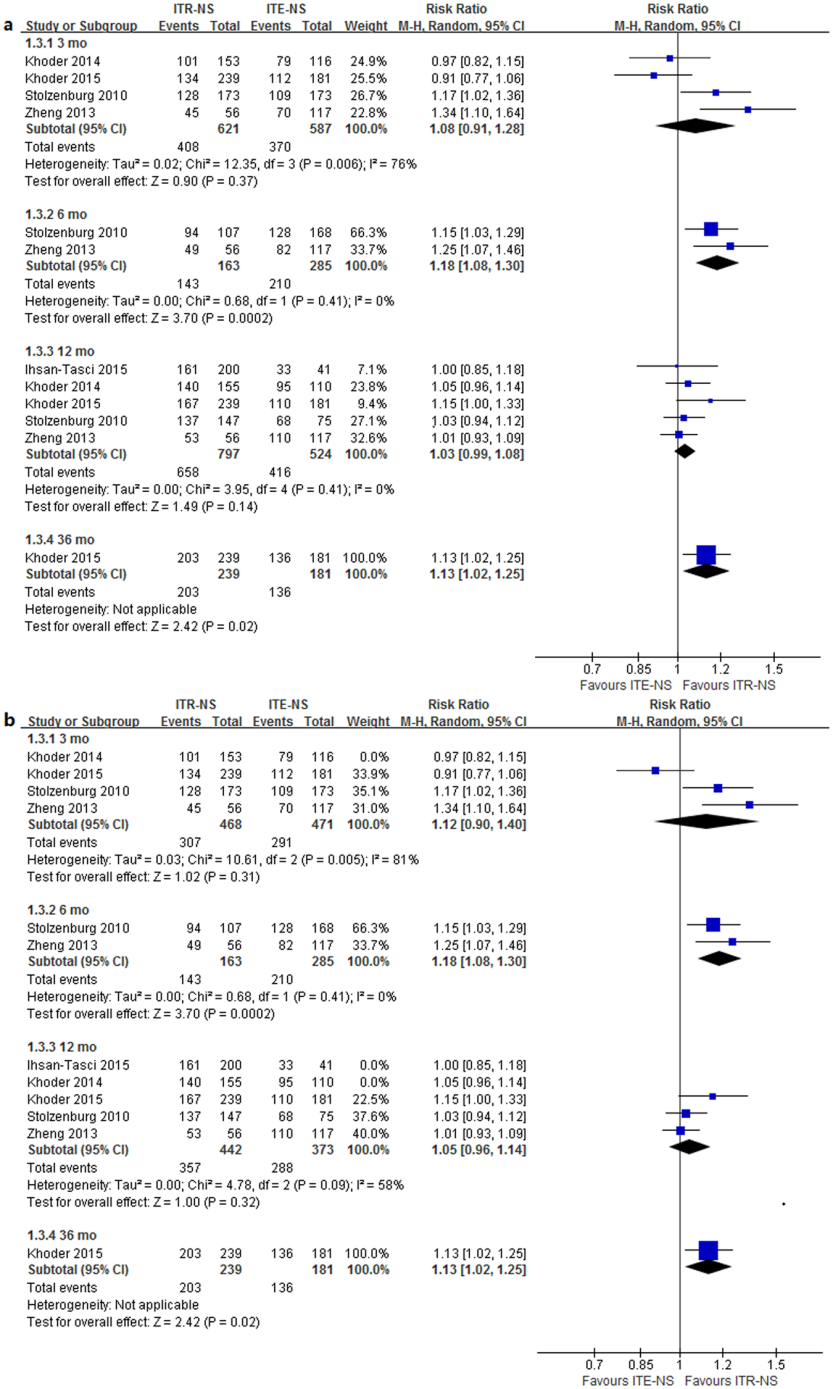
Forest plot of (**a**) continence rates (**b**) sensitivity analysis for ITR-NS versus ITE-NS. CI = confidence interval; ITR-NS = intrafascial nerve sparing; ITE-NS = interfascial nerve sparing; MH = Mantel-Haenszel.

Four studies^7, 9, 10, 12^ reported the postoperative urinary continence recovery rate at 3 mo; two studies^7, 9^ reported it at 6 mo; five studies^7, 9–12^ reported it at 12 mo; one study^12^ reported it at 36 mo (Table 5). Heterogeneity was detected in the 3 mo time point (*p* = 0.006, I^2^ = 76%). The results of meta-analysis with random-effects model showed that patients undergoing ITR-NS had significantly better continence outcomes reported on 6 mo (RR = 1.18, 95% CI 1.08–1.30, *p* = 0.0002) and 36 mo (RR = 1.13, 95% CI 1.02–1.25, *p* = 0.02) compared with those undergoing ITE-NS (Fig. 2a). No significant difference was found at 3 mo (RR = 1.08, 95% CI 0.91–1.28; *p* = 0.37) and 12 mo (RR = 1.03, 95% CI 0.99–1.08; *p* = 0.14) (Fig. 2a). Sensitivity analysis showed similar results to overall analysis (Fig. 2b). The quality of evidence was very low for continence recovery at different timing (Supplementary Table 3).

**Table 5:** Continence recovery in the studies comparing intrafacial and interfacial nerve-sparing radical prostatectomy.

Study	No. of cases, type	Method	Criterion	3 mo, %	6 mo, %	12 mo, %	36 mo, %
Stolzenburg^7^	200 ITR-NS	ICS	0–1 pads/d	74	87.9	93.2	—
	200 ITE-NS			63	76.2	90	—
Zheng^9^	65 ITR-NS	Questionnaire	0–1 pads/d	80.4	87.5	96.6	—
	130 ITE-NS			59.8	70.1	94	—
Khoder^10^	203 ITR-NS	Questionnaire	0 pad/d	66	—	90	—
	163 ITE-NS			68	—	86	—
Ihsan-Tasci^11^	200 ITR-NS	Not described	Only safety pads used	—	—	80.5	—
	41 ITE-NS			—	—	80.4	—
Khoder^12^	239 ITR-NS	Questionnaire	0 pad/d	56	—	70	85
	181 ITE-NS			62	—	61	75

ITR-NS = intrafacial nerve sparing; ITE-NS = interfacial nerve sparing; ICS = International Continence Society.

#### Erectile function

**Figure 3:**
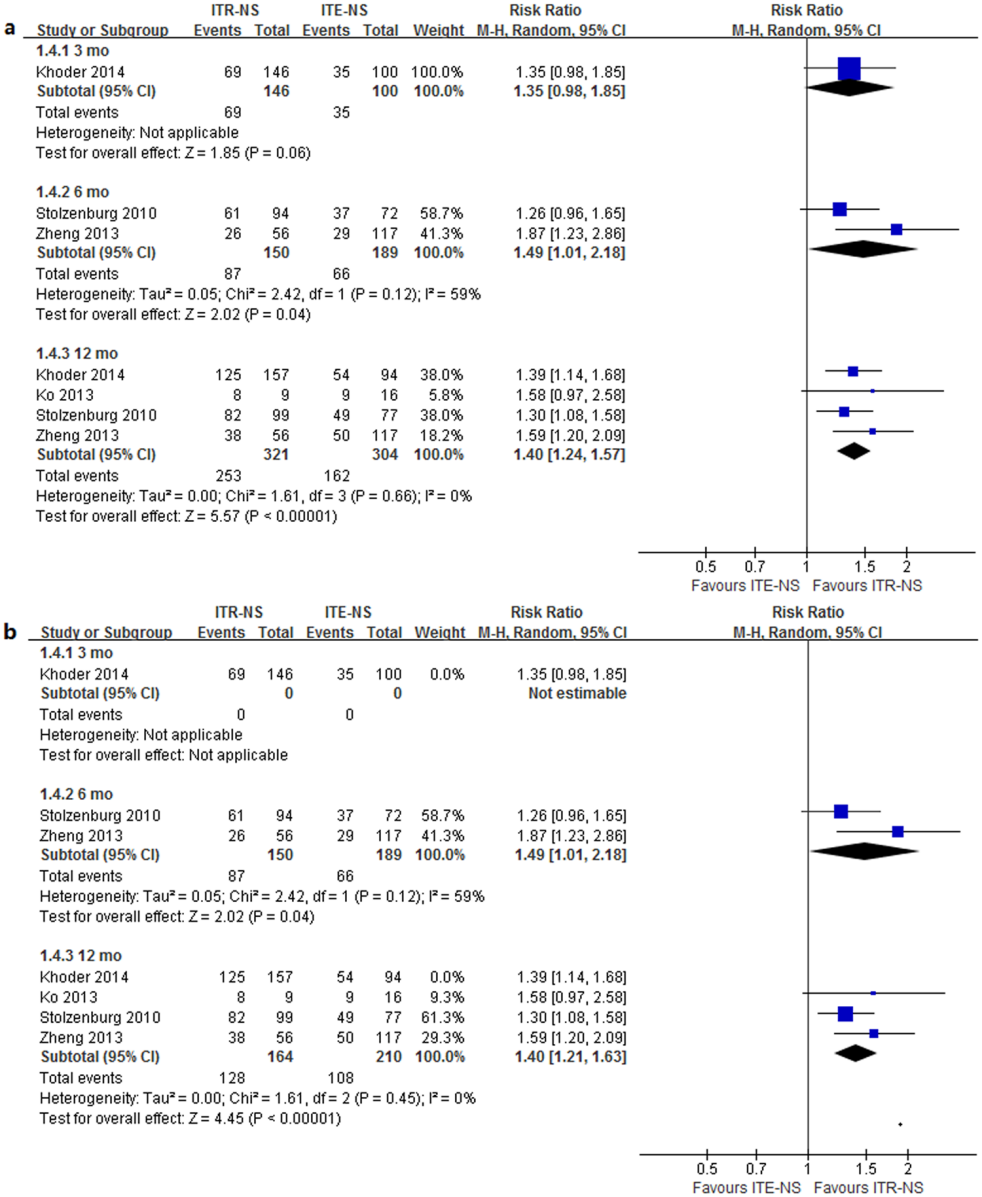
Forest plot of (**a**) potency rates (**b**) sensitivity analysis for ITR-NS versus ITE-NS. CI = confidence interval; ITR-NS = intrafascial nerve sparing; ITE-NS = interfascial nerve sparing; MH = Mantel-Haenszel.

One study^10^ reported the postoperative potency recovery rate at 3 mo; two studies^7, 9^ reported it at 6 mo; four studies^7–10^ reported it at 12 mo (Table 6). Heterogeneity was detected in the 6 mo time point (*p* = 0.04, I^2^ = 59%). The results of meta-analysis with random-effects model showed that patients undergoing ITR-NS had significantly better potency outcomes reported on 6 mo (RR = 1.49, 95% CI 1.01–2.18, *p* = 0.04) and 12 mo (RR = 1.40, 95% CI 1.24–1.57, *p* < 0.00001) compared with those suffering from ITE-NS (Fig. 3a). No significant difference was found at 3 mo (RR = 1.35, 95% CI 0.98–1.85, *p* = 0.06) (Fig. 3a). Sensitivity analysis showed similar results to overall analysis (Fig. 3b). The quality of evidence was very low for potency at different timing (Supplementary Table 3).

**Table 6:** Erectile function in the studies comparing intrafacial and interfacial nerve-sparing radical prostatectomy.

Study	No. of cases, type	Method	Criterion	3 mo, %	6 mo, %	12 mo, %
Stolzenburg^7^	200 ITR-NS	IIEF and SEP	Erectile function sufficient for intercourse with or without the help of PDE-5 inhibitors	—	64.8	82.8
	200 ITE-NS			—	51.4	64.8
Ko^8^	9 ITR-NS	Questionnaire	Erections adequate for vaginal penetration with satisfaction, with or without the use of a PDE-5 inhibitor	—	—	88.9
	32ITE-NS			—	—	65.6
Zheng^9^	65 ITR-NS	SHIM	Total scores of ≥22 in the SHIM questionnaire	—	46.4	67.9
	130 ITE-NS			—	24.8	42.7
Khoder^10^	203 ITR-NS	IIEF-5	Patients achievement of a composite score of 15 points or higher on the IIEF-5 questionnaire	47	—	80
	163 ITE-NS			35	—	57

ITR-NS = intrafacial nerve sparing; ITE-NS = interfacial nerve sparing; IEEF = International Index of Erectile Function; SEP = Sexual Encounter Profile diaries; SHIM = Sexual Health Inventory for Men questionnaire; PDE-5 = phosphodiesterase type 5.

#### PSM

**Figure 4:**
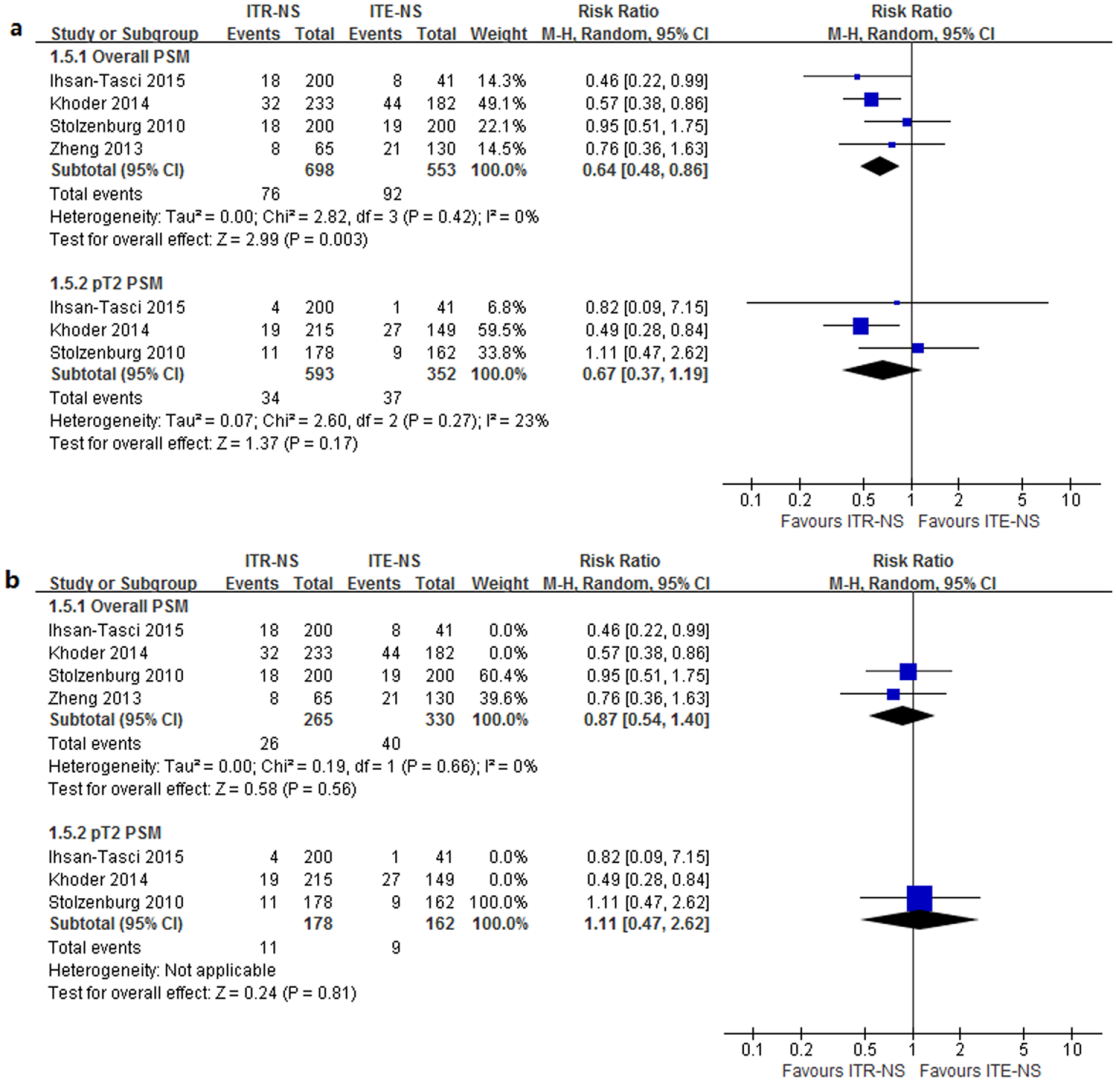
Forest plot of (**a**) PSM and pT2 PSM (**b**) sensitivity analysis for ITR-NS versus ITE-NS. CI = confidence interval; ITR-NS = intrafascial nerve sparing; ITE-NS = interfascial nerve sparing; MH = Mantel-Haenszel; PSM = positive surgical margin.

Four studies^7, 9–11^ reported the PSM rate after the RP (Table 7). No evidence of heterogeneity was found between the studies (*p* = 0.42, I^2^ = 0%). The results of meta-analysis with random-effects model showed that patients undergoing ITR-NS had significantly lower PSM rate compared with those experiencing ITE-NS (RR = 0.64, 95% CI 0.48–0.86, *p* = 0.003; Fig. 4a). Sensitivity analysis showed that the significant difference was disappeared when retrospective studies were excluded (Fig. 4b). In ITR-NS compared with ITE-NS, RR was 0.87 (0.54–1.40, *p* = 0.56; Fig. 4b) for PSM rate. The quality of evidence was very low for PSM (Supplementary Table 3).

**Table 7:** Positive surgical margin (PSM) rates in the studies comparing intrafacial and interfacial nerve-sparing radical prostatectomy.

Study	No. of cases, type	Overall PSM, %	pT2 PSM, %
Stolzenburg^7^	200 ITR-NS	9	6.2
	200 ITE-NS	9.5	5.6
Zheng^9^	65 ITR-NS	12.3	—
	130 ITE-NS	16.2	—
Khoder^10^	203 ITR-NS	13.7	8.8
	163 ITE-NS	24.2	18.1
Ihsan-Tasci^11^	200 ITR-NS	9	1.2
	41 ITE-NS	19.5	0.3

ITR-NS = intrafacial nerve sparing; ITE-NS = interfacial nerve sparing; PSM = positive surgical margin.

#### pT2 PSM

Three^7, 10, 11^ studies reported the pT2 PSM rate after the RP (Table 7). Low to moderate between-study heterogeneity was detected (*p* = 0.27, I^2^ = 23%). The results of meta-analysis with random-effects model showed that patients receiving ITR-NS had similar pT2 PSM rate compared with those undergoing ITE-NS (RR = 0.67, 95% CI 0.37–1.19, *p* = 0.17; Fig. 4a). Sensitivity analysis showed similar results to overall analysis (Fig. 4b). The quality of evidence was very low for pT2 PSM (Supplementary Table 3).

#### BCR free rates

**Figure 5:**
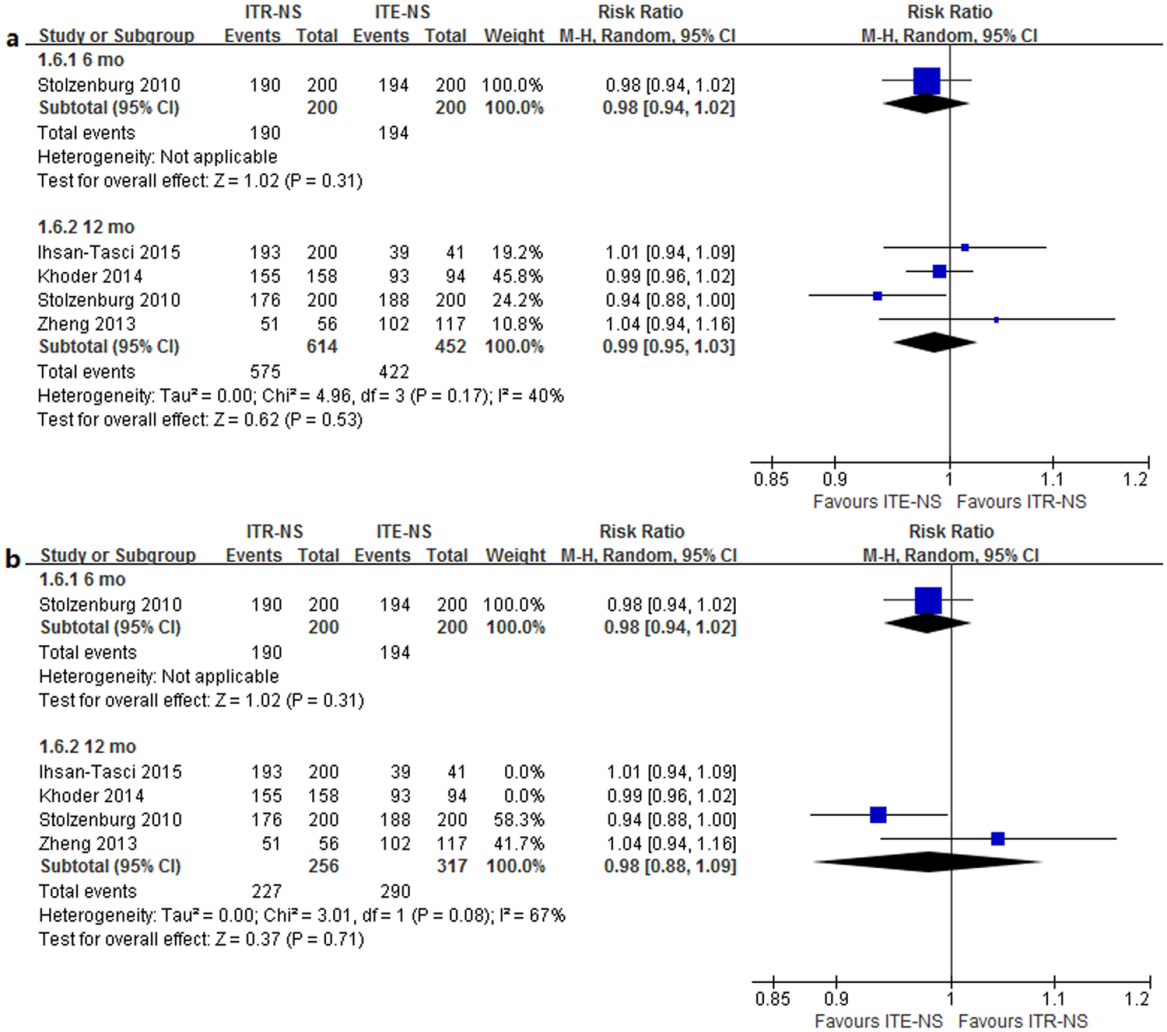
Forest plot of (**a**) BCR free rates (**b**) sensitivity analysis for ITR-NS versus ITE-NS. BCR = biochemical recurrence; CI = confidence interval; ITR-NS = intrafascial nerve sparing; ITE-NS = interfascial nerve sparing; MH = Mantel-Haenszel.

One study^7^ reported the BCR free rates at 6 mo and four studies^7, 9–11^ reported them at 12 mo (Table 8). Moderate between-study heterogeneity was detected in the 12 mo point (*p* = 0.17, I^2^ = 40%). The results of meta-analysis with random-effects model showed that patients undergoing ITR-NS had similar BCR free rate compared with those experiencing ITE-NS (6 mo: RR = 0.98, 95% CI 0.94–1.02, *p* = 0.31; 12 mo: RR = 0.99, 95% CI 0.95–1.03, *p* = 0.53; Fig. 5a). Sensitivity analysis showed similar results to overall analysis (Fig. 5b). The quality of evidence was very low for BCR free rates at different timing (Supplementary Table 3).

**Table 8:** Biochemical free rates in the studies comparing intrafacial and interfacial nerve-sparing radical prostatectomy.

Study	No. of cases, type	Criterion	6 mo, %	12 mo. %
Stolzenburg^7^	200 ITR-NS	PSA ≤ 0.1 ng/ml	95.2	87.8
	200 ITE-NS		96.9	93.9
Zheng^9^	65 ITR-NS	Not described	—	91.1
	130 ITE-NS		—	87.2
Khoder^10^	203 ITR-NS	Not described	—	98.1
	163 ITE-NS		—	98.9
Ihsan-Tasci^11^	200 ITR-NS	PSA ≤ 0.2 ng/ml	—	96.5
	41 ITE-NS		—	95.1

ITR-NS = intrafacial nerve sparing; ITE-NS = interfacial nerve sparing; PSA = prostate specific antigen.

#### Secondary outcomes (perioperative parameters)

**Figure 6:**
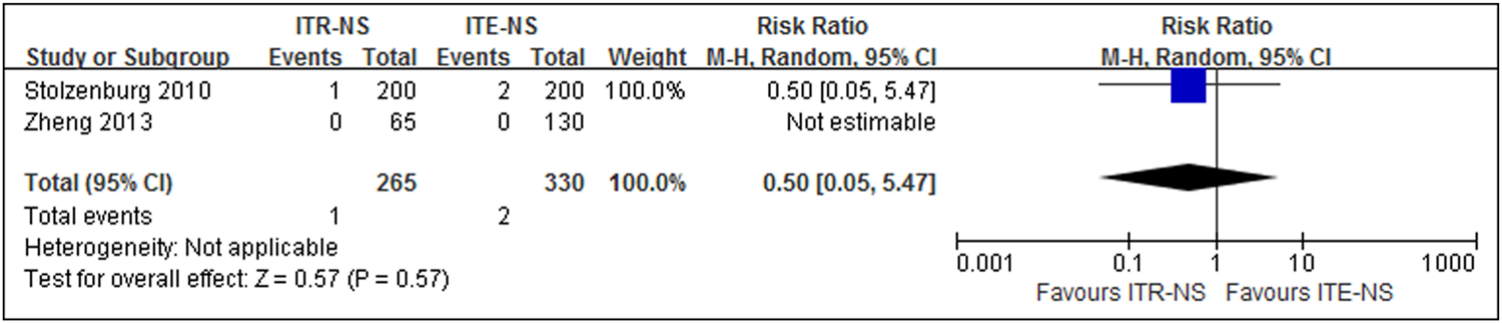
Forest plot of transfusion rates for ITR-NS versus ITE-NS. CI = confidence interval; ITR-NS = intrafascial nerve sparing; ITE-NS = interfascial nerve sparing; MH = Mantel-Haenszel.

The perioperative parameters are presented in Table 9. Two studies^7, 9^ reported the transfusion rate. The results of meta-analysis with random-effects model showed that patients suffering from ITR-NS had similar transfusion rates compared with those undergoing ITE-NS (RR = 0.50, 95% CI 0.05–5.47, p = 0.57; Fig. 6). The mean operation time ranged from 60^10^ to 169.41 min^8^. The mean blood loss ranged from 87^9^ to 200 ml^7^. The mean duration of catheterization ranged from 5^7^ to 11.09 d^8^. The mean hospital stay was 8 d^9^.

**Table 9:** Perioperative parameters in the studies comparing intrafacial and interfacial nerve-sparing radical prostatectomy.

Study	No. of cases, type	Operative time, min	Blood loss, ml	Transfusion rates, %	Duration of catheterization, d	Hospital stay, d
Stolzenburg^7^	200 ITR-NS	140 (70–280)	200 (30–1100)	0.5	6 (5–20)	—
	200 ITE-NS	135 (50–250)	200 (20–800)	1	5 (3–20)	—
Ko^8^	9 ITR-NS	157.78 ± 33.83	138.89 ± 79.17	—	9.67 ± 1.66	—
	32 ITE-NS	169.41 ± 43.01	175.78 ± 128.34	—	11.09 ± 2.70	—
Zheng^9^	65 ITR-NS	100 (89–106)	94 (81–98)	0	7 (6–8)	8 (8–9)
	130 ITE-NS	96 (86–104)	87 (75–100)	0	7 (6–9)	8 (7–10)
Khoder^10^	203 ITR-NS	60 (40–120)	100 (50–600)	—	—	—
	163 ITE-NS	65 (45–195)	150 (50–900)	—	—	—
Khoder^12^	239 ITR-NS	65 (40–200)	100 (50–800)	—	—	—
	181 ITE-NS	65 (45–215)	150 (50–1300)	—	—	—

ITR-NS = intrafacial nerve sparing; ITE-NS = interfacial nerve sparing.

### Publication bias

**Figure 7:**
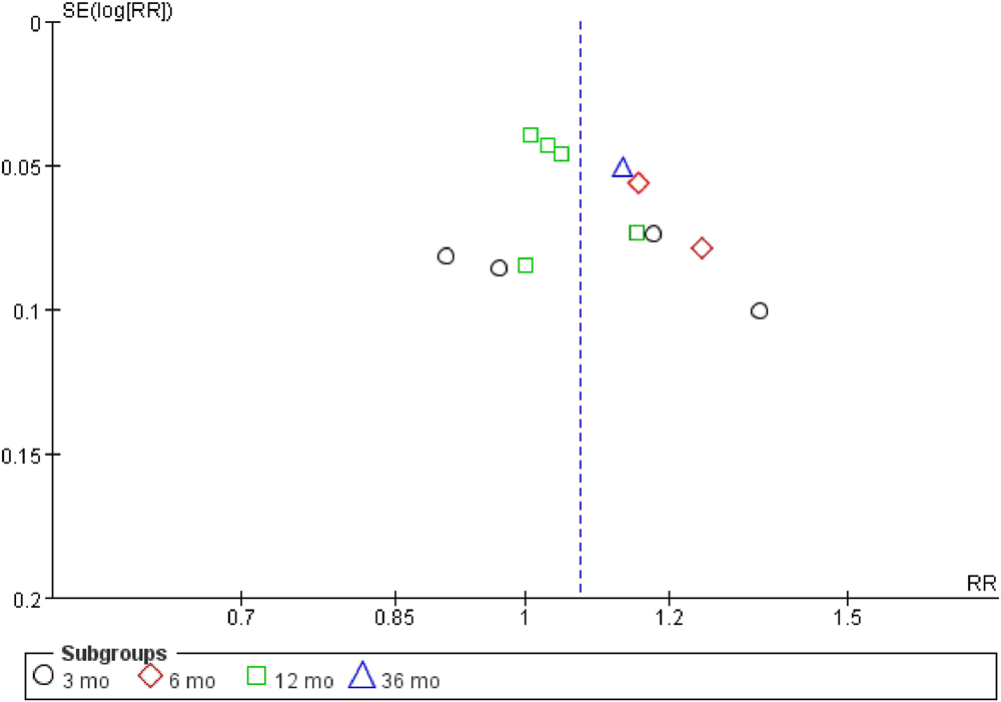
Funnel plot of urinary continence recovery rate.

Publication bias was detected only for continence recovery. The result of funnel plot provided certain evidence that publication bias existed (Fig. 7).

## Discussion

In this systematic review and meta-analysis of six studies, we compared the effectiveness and safety of ITR-NS and ITE-NS on prostate cancer treatment. Irrespective of the surgical technique, we found that ITR-NS had better functional results (urinary continence and erectile function) and oncologic outcome (PSM, pT2 PSM, and BCR) compared with ITE-NS. These findings were supported by sensitivity analyses which took the prospective studies into consideration alone. The results suggested that there was a difference in continence between techniques at 6 months and 36 months but not at 12 months. This might be caused by the various procedures of different techniques or it was a spurious result.

To our knowledge, this systematic review and meta-analysis is the first study to comprehensively evaluate this topic. The previous reviews or systematic reviews or meta-analysis evaluated the techniques of RP^5, 18–20^ (such as RARP, laparoscopic, and retropubic open), the PSM and perioperative complication rates of primary surgical treatments^21^, the primary surgical treatments for prostate cancer^22–26^, transperitoneal and extraperitoneal robot-assisted RP^27^, and the efficacy and safety of conventional laparoscopic RP with a transperitoneal approach versus that of an extraperitoneal approach^28^. Therefore, none of these studies focused on the surgical technique of RP. In 2015, Reeves *et al*.^6^ systematically reviewed the association of NVBs sparing in RP with postoperative urinary continence outcomes. They found that avoiding damage to the nerve activity surrounding the prostate promotes urinary control in the first 6 mo after nerve sparing RP. In addition, from theoretically, the ITR-NS had better function than ITE-NS in functional outcomes as we mentioned in introduction. The result of our systematic review and meta-analysis was supported by the Reeves’s study^6^.

Tewari *et al*.^29^ proposed a grading system based on four grades of dissection according to veins surrounding the prostate. Schatloff *et al*.^30^ proposed a grading system based on five grades of dissection according to arterial periprostatic vasculature. The grade 1 of Tewari’s approach and grade 5 of Schatloff’s approach was equal to ITR-NS. As we acknowledged that cancer control is the most important goal of RP. The different dissection planes concept aims for an incremental security margin of prostate, instead of true incremental nerves sparing^13^. In this review, we found that ITR-NS was not significantly presented with risk of PSM, pT2 PSM and BCR free rate compared with ITR-NS. This might be due to restricted patient selection of the included studies. Therefore, the choice of surgical technique or dissection plane should be made based on the specific situation of patients in clinical practice, such as clinical examination, biopsy results, and imaging results^6^.

Although we used a systematic method to perform the meta-analysis, certain limitations also should be taken into consideration. First, our systematic review only identified one RCT, and the absence of high quality RCT might weaken the reliability of the meta-analysis. Second, low to moderate between-study heterogeneity was detected, which might be attributed to different surgical techniques, study design, selection bias, and surgeon experience. Selection bias between the two techniques was a major bias in the present meta-analysis, which implied that higher risk patients tended to undergo interfascial technique and lower risk patients tended to an intrafascial technique. However, patients with Gleason score more than 8 were only in two trials and the PSA levels were all similar as presented in Table 2. In addition, the sensitivity analysis also showed similar results to the overall analysis. Therefore, the selection bias was not obvious in this systematic review. Two studies^7, 9^ used laparoscopic RP; two studies^8, 11^ used robot-assisted RP; and two studies^10, 12^ used open retropubic RP. These six comparative trials included one RCT^7^, three prospective comparative trials^8, 9, 12^, and two retrospective comparative trials^10, 11^. We performed sensitivity analysis through excluding retrospective studies. The summary result of PSM rate was changed while excluding the retrospective studies. Therefore, the robustness of the result is weak. Third, we only included studies published in English. In addition, grey literature was not included. Hence, language bias might occur in this study. Fourth, due to limited number of included studies, we did not fully detect the publication bias. Of course, the publication bias is inevitable because we included studies published in English and excluded grey literature. The publication bias might decrease the reliability and credibility of this meta-analysis and systematic review. Moreover, the sample size and statistical power were relatively insufficient to identify the true difference of the two surgical techniques. Ultimately, the meta-analysis is a secondary analysis and its quality is based on the included studies. Our meta-analysis included studies with a RCT with high risk of bias and five longitudinal studies with moderate risk of bias. Therefore, the quality of the evidence was consequentially degraded.

With respect to further researches, multi-center clinical trials, if possible, RCTs should be performed to evaluate the effectiveness and safety of ITR-NS and ITE-NS. In addition, further studies also should elucidate the functional anatomy of urinary continence and erectile function.

In conclusion, this systematic review and meta-analysis demonstrates that ITR-NS has better continence at 6 mo and 36 mo and better potency recovery at 6 mo and 12 mo postoperatively, regardless of the surgical technique. This finding might be due to more nerves were saved and less damage of the periprostatic tissue in ITR-NS compared with ITE-NS. The cancer control of ITR-NS was also better than that of ITE-NS. This may be explained by the fact that patients in ITE-NS group present higher risk cancer than patients in ITR-NS group. Further studies are needed to verify the conclusion in future.

## Electronic supplementary material

Supplementary tables
