# Supplementary material for: Intrafascial versus interfascial nerve sparing in radical prostatectomy for localized prostate cancer: a systematic review and meta-analysis
Source: Sci Rep. 2017 Sep 13;7:11454. doi: 10.1038/s41598-017-11878-7 (PMC5597634; doi:10.1038/s41598-017-11878-7)
Supplement: Supplementary file 1 — Supplementary tables [file 41598_2017_11878_MOESM1_ESM.pdf]

Intrafascial versus interfascial nerve sparing in radical prostatectomy for localized prostate cancer: a systematic review and meta-analysis

Hong Weng[1,2*], Xian-Tao Zeng[1,2*], Sheng Li[1], Xiang-Yu Meng[1], Ming-Jun Shi[3], Da-Lin He[4], Xing-Huan Wang[1,2]

[1]Center for Evidence-Based and Translational Medicine, Zhongnan Hospital of Wuhan University, Wuhan 430071, China.

[2]Department of Urology, Zhongnan Hospital of Wuhan University, Wuhan 430071, China.

[3]Institut Curie, Centre National de la Recherche Scientifique (CNRS), Unité Mixte de Recherche 144, Paris 75248, France.

[4]Department of Urology, The First Affiliated Hospital of Xi'an Jiaotong University, Xi'an 710061.

[*]These authors contributed equally to this work.

Correspondence to: Xing-Huan Wang, Center for Evidence-Based and Translational Medicine, Zhongnan Hospital of Wuhan University, 169 Donghu Road, Wuchang District, Wuhan 430071, Hubei Province, P.R. China. E-mail: wangxinghuan1965@163.com, wangxinghuan@whu.edu.cn; Tel: +86 027 6781 2817, Fax: +86 027 6781 2817.

Supplementary Table 1 – Search strategy and results of each database

No.	Query	Results
**PubMed**		
#14	#11 AND #12 AND #13	66
#13	#9 OR #10	261
#12	#6 OR #7 OR #8	33868
#11	#1 OR #2 OR #3 OR #4 OR #5	168385
#10	intrafascial[Text Word]	173
#9	interfascial[Text Word]	107
#8	"radical prostatectomy"[Text Word]	15593
#7	prostatectomy[Text Word]	33869
#6	prostatectomy[MeSH Terms]	26064
#5	"prostate cancer"[text word]	84391
#4	prostate[Text Word]	152514
#3	prostate[MeSH Terms]	30726
#2	"prostatic neoplasms"[Text Word]	102575
#1	"prostatic neoplasms"[MeSH Terms]	102264
**Embase**		
#11	#8 AND #9 AND #10	148
#10	#6 OR #7	464
#9	#4 OR #5	50127
#8	#1 OR #2 OR #3	263614
#7	intrafascial	312
#6	interfascial	195
#5	'radical prostatectomy'	25592
#4	'prostatectomy'	50127
#3	'prostate cancer'	161212
#2	prostate	263506
#1	'prostatic neoplasms'/exp OR 'prostatic neoplasms'	183460

Cochrane Library

#4	#1 and #2 and #3	2
#3	“interfascial” or "intrafascial"	16
#2	"prostatectomy" or "radical prostatectomy"	2444
#1	"prostatic neoplasms" or "prostate" or "prostate cancer"	12480

Supplementary Table 2 – Definition of the ITR-NS and ITE-NS.

Studies	Definition of ITR-NS	Definition of ITE-NS
Stolzenburg, 2010	Dissecting the plane between prostatic capsule and prostatic fascia	Dissecting the plane between prostatic fascia and endopelvic fascia
Ko, 2013	ITR-NS implies lateral prostatic fascia preservation, developing the plane between the prostatic fascia and NVB and incising high anteriorly over the prostate	ITE-NS is associated with dissection into the avascular plane between the prostate fascia (so called ‘prostatic capsule’) and Denonvilliers’ fascia to preserve the NVB
Zheng, 2013	Dissecting the plane between prostatic capsule and prostatic fascia	Dissecting the plane between prostatic fascia and endopelvic fascia
Khoder, 2014	Endopelvic fascia is not incised and the puboprostatic ligaments are preserved or cut just proximal to prostate with lateralisation landing in a plan between the prostate capsule and levator ani fascia	The endopelvic fascia is opened laterally. The Denonvillier’s fascia is cut laterally to develop the interfascial plain preserving the NVB dorsal to the prostate
Ihsan-Tasci, 2015	Dissecting the plane between prostatic capsule and prostatic fascia	Dissecting the plane between prostatic fascia and endopelvic fascia
Khoder, 2015	It involves small incision of the endopelvic fascia only ventrally medial to puboprostatic ligaments which are cut just proximal to prostate opening the intrafascial plan	This technique involves the incision of endopelvic fascia, incising the levator and prostatic fasciae at high lateral positions over the prostate to developing the plane between the prostatic capsule and prostatic fascia

ITR-NS: intrafascial nerve-sparing; ITE-NS: interfascial nerve-sparing; NVB, neurovascular bundles.

Supplementary Table 3 – The quality of the evidence assessment of the outcomes.

Quality assessment							№ of patients		Effect		Quality	Importance
№ of studies	Study design	Risk of bias	Inconsistency	Indirectness	Imprecision	Other considerations	ITR-NS	ITE-NS	Relative (95% CI)	Absolute (95% CI)		
Continence recovery - 3 mo												
4	observational studies	not serious	very serious [a]	not serious	not serious	publication bias strongly suspected [b]	408/621 (65.7%)	370/587 (63.0%)	**RR 1.08** (0.91 to 1.28)	**50 more per 1,000** (from 57 fewer to 176 more)	⊕◯◯◯ VERY LOW	CRITICAL
Continence recovery - 6 mo												
2	observational studies	not serious	not serious	not serious	not serious	publication bias strongly suspected [b]	143/163 (87.7%)	210/285 (73.7%)	**RR 1.18** (1.08 to 1.30)	**133 more per 1,000** (from 59 more to 221 more)	⊕◯◯◯ VERY LOW	CRITICAL
Continence recovery - 12 mo												
5	observational studies	not serious	not serious	not serious	not serious	publication bias strongly suspected [b]	658/797 (82.6%)	416/524 (79.4%)	**RR 1.03** (0.99 to 1.08)	**24 more per 1,000** (from 8 fewer to 64 more)	⊕◯◯◯ VERY LOW	CRITICAL
Continence recovery - 36 mo												
1	observational studies	not serious	not serious	not serious	not serious	publication bias strongly suspected [b]	203/239 (84.9%)	136/181 (75.1%)	**RR 1.13** (1.02 to 1.25)	**98 more per 1,000** (from 15 more to 188 more)	⊕◯◯◯ VERY LOW	CRITICAL
Potency - 3 mo												

Quality assessment							№ of patients		Effect		Quality	Importance
**№ of studies**	**Study design**	**Risk of bias**	**Inconsistency**	**Indirectness**	**Imprecision**	**Other considerations**	**ITR-NS**	**ITE-NS**	**Relative (95% CI)**	**Absolute (95% CI)**		
1	observational studies	not serious	not serious	not serious	not serious	publication bias strongly suspected [b]	69/146 (47.3%)	35/100 (35.0%)	**RR 1.35** (0.98 to 1.85)	**123 more per 1,000** (from 7 fewer to 298 more)	⨁◯◯◯ VERY LOW	CRITICAL
Potency - 6 mo												
2	observational studies	not serious	serious [c]	not serious	not serious	publication bias strongly suspected [b]	87/150 (58.0%)	66/189 (34.9%)	**RR 1.49** (1.01 to 2.18)	**171 more per 1,000** (from 3 more to 412 more)	⨁◯◯◯ VERY LOW	CRITICAL
Potency - 12 mo												
4	observational studies	not serious	not serious	not serious	not serious	publication bias strongly suspected [b]	253/321 (78.8%)	162/304 (53.3%)	**RR 1.40** (1.24 to 1.57)	**213 more per 1,000** (from 128 more to 304 more)	⨁◯◯◯ VERY LOW	CRITICAL
PSM - Overall PSM												
4	observational studies	not serious	not serious	not serious	not serious	publication bias strongly suspected [b]	76/698 (10.9%)	92/553 (16.6%)	**RR 0.64** (0.48 to 0.86)	**60 fewer per 1,000** (from 23 fewer to 87 fewer)	⨁◯◯◯ VERY LOW	CRITICAL
PSM - pT2 PSM												

Quality assessment							№ of patients		Effect		Quality	Importance
№ of studies	Study design	Risk of bias	Inconsistency	Indirectness	Imprecision	Other considerations	ITR-NS	ITE-NS	Relative (95% CI)	Absolute (95% CI)		
3	observational studies	not serious	not serious [b]	not serious	not serious	publication bias strongly suspected [b]	34/593 (5.7%)	37/352 (10.5%)	**RR 0.67** (0.37 to 1.19)	**35 fewer per 1,000** (from 20 more to 66 fewer)	⨁◯◯◯ VERY LOW	CRITICAL
Biochemical free rates - 6 mo												
1	randomised trials	not serious	not serious	not serious	not serious	publication bias strongly suspected [b]	190/200 (95.0%)	194/200 (97.0%)	**RR 0.98** (0.94 to 1.02)	**19 fewer per 1,000** (from 19 more to 58 fewer)	⨁⨁⨁◯ MODERATE	IMPORTANT
Biochemical free rates - 12 mo												
4	observational studies	not serious	serious [c]	not serious	not serious	publication bias strongly suspected [b]	575/614 (93.6%)	422/452 (93.4%)	**RR 0.99** (0.95 to 1.03)	**9 fewer per 1,000** (from 28 more to 47 fewer)	⨁◯◯◯ VERY LOW	IMPORTANT

CI: Confidence interval; **RR:** Risk ratio; **MD:** Mean difference

a. The between-study heterogeneity was high
b. Publication bias was strongly suspected
c. The between-study heterogeneity was moderate
